# Post-operative atrial fibrillation examined using whole-genome RNA sequencing in human left atrial tissue

**DOI:** 10.1186/s12920-017-0270-5

**Published:** 2017-05-02

**Authors:** Martin I Sigurdsson, Louis Saddic, Mahyar Heydarpour, Tzuu-Wang Chang, Prem Shekar, Sary Aranki, Gregory S Couper, Stanton K. Shernan, Jochen D. Muehlschlegel, Simon C. Body

**Affiliations:** 1 Department of Anesthesiology, Perioperative and Pain Medicine, Brigham and Women’s Hospital/Harvard Medical School, 75 Francis Street, Boston, MA 02115 USA; 2 Division of Cardiac Surgery, Department of Surgery, Brigham and Women’s Hospital/Harvard Medical School, Boston, MA USA

**Keywords:** Atrial fibrillation, Mitral valve surgery, eQTL, Expression quantitative trait loci

## Abstract

**Background:**

Both ambulatory atrial fibrillation (AF) and post-operative AF (poAF) are associated with substantial morbidity and mortality. Analyzing the tissue-specific gene expression in the left atrium (LA) can identify novel genes associated with AF and further the understanding of the mechanism by which previously identified genetic variants associated with AF mediate their effects.

**Methods:**

LA free wall samples were obtained intraoperatively immediately prior to mitral valve surgery in 62 Caucasian individuals. Gene expression was quantified on mRNA harvested from these samples using RNA sequencing. An expression quantitative trait loci (eQTL) analysis was performed, comparing gene expression between different genotypes of 1.0 million genetic markers, emphasizing genomic regions and genes associated with AF.

**Results:**

Comparison of whole-genome expression between patients who later developed poAF and those who did not identified 23 differentially expressed genes. These included genes associated with the resting membrane potential modified by potassium currents, as well as genes within Wnt signaling and cyclic GMP metabolism. The eQTL analysis identified 16,139 *cis* eQTL relationships in the LA, including several involving genes and single nucleotide polymorphisms (SNPs) linked to AF. A previous relationship between rs3744029 and *MYOZ1* expression was confirmed, and a novel relationship between rs6795970 and the expression of the *SCN10A* gene was identified.

**Conclusions:**

The current study is the first analysis of the human LA expression landscape using high-throughput RNA sequencing. Several novel genes and variants likely involved in AF pathogenesis were identified, thus furthering the understanding of how variants associated with AF mediate their effects via altered gene expression.

**Trial registration:**

ClinicalTrials.gov ID: NCT00833313, registered 5. January 2009

**Electronic supplementary material:**

The online version of this article (doi:10.1186/s12920-017-0270-5) contains supplementary material, which is available to authorized users.

## Background

The human left atrium (LA) has unique tissue and electrophysiological characteristics. It is associated with the pathogenesis of diseases in the conduction system, most notably atrial fibrillation (AF). To date, multiple genetic variants have been associated with AF, including variants in the 4q25 locus containing the paired-like homeodomain 2 (*PITX2*) gene associated with the development of pulmonary vasculature [1], the 1q21 locus containing the member 3 potassium intermediate/small conductance calcium-activated channel member 3 (*KCCN3*) gene involved in cardiac conductance [2], and the 16q22 locus containing the zinc finger homeobox 3 (*ZFHX3*) gene with unknown functional significance [3]. Furthermore, variants within genes associated with ion conductance, such as genes encoding potassium and sodium channels, have been associated with AF [4–6]. Moreover, variants in 4q25 and 1q21 have also been associated with post-operative atrial fibrillation (poAF) after cardiac surgery [7, 8], indicating that the genetic background underlying both ambulatory AF and poAF may be similar.

Previous studies examining the genetic variants associated with AF do not address their association with tissue-specific expression changes in the human LA that can contribute to the pathophysiology of AF. Comparing tissue-specific gene expression between sequence variants with expression quantitative loci (eQTL) analysis [9], can identify new variants associated with AF and further the understanding of how variants associated with AF mediate their effects.

Here, we used high throughput RNA sequencing to compare the gene expression pattern in the LA between those patients who did and did not develop poAF. Furthermore, we explored the genome-wide relationships between DNA sequence variants and whole-genome RNA expression of the LA, with a focus on regions and genes previously associated with AF and poAF.

## Methods

### Patients

Sixty three patients with self-reported two-generation Caucasian ancestry undergoing mitral valve repair or replacement between 12/2012 and 4/2014 were recruited for this study. We obtained LA tissue from the upper border of the routine left atriotomy incision in close proximity to the left pulmonary veins. The study was approved by the Institutional Review Board and all patients provided written informed consent.

Demographics were collected for all patients as well as data pertaining to the cardiac procedure. Images acquired from routine intraoperative transesophageal echocardiography were used to calculate LA volume normalized by body surface area using the ellipsoidal formula [10]. All patients were in sinus rhythm at the day of surgery. Patients were prospectively followed during the hospital stay for multiple clinical outcomes, including poAF. Similar to prior studies [7, 8], poAF was defined as any AF diagnosed by a clinician that developed during the primary hospitalization. Patients were monitored via continuous telemetry throughout their hospitalization.

### Sequencing and genotyping

After harvesting, tissue samples were stored in RNAlater (Ambion; ThermoFisher Scientific) solution and whole RNA extraction was done by standardized methods. After reverse transcription of single-stranded RNA to double-stranded DNA, isolation of short fragments, and poly(A) addition and ligation of adaptors, 90 base-pair paired-end sequencing was performed on an Illumina HiSeq 2000 (Illumina, San Diego, CA). Prior to data analysis, adapter sequences were trimmed and low-quality reads were removed.

Using DNA isolated from whole blood, single nucleotide polymorphism (SNP) genotyping of 63 patients was performed using the Illumina Omni2.5 with exome content genotyping array (Illumina, San Diego, CA). One patient’s DNA failed QC (genotyping call rate <98%) and was excluded from further analysis.

To test whether SNPs with eQTL association with differentially expressed genes in the LA of patients with poAF were associated with poAF in a larger cohort, we used a second cohort of 1,805 patients undergoing heart surgery at BWH [7, 8]. DNA extracted from peripheral blood of study participants was used to genotype eight SNPs using the iPLEX Gold method on a Sequenom Mass Array system (Sequenom, San Diego, CA). Genotypes were called with the TypeAnalyzer Application.

### Alignment and Annotation

Sequenced reads were aligned to the UCSC hg19 version of the human genome using TopHat2 (v2.0.9) [11] from the Bowtie2 algorithm [12]. Default parameters were used for the alignments except for mate inner distance and mate standard deviation, which were set at 165 and 37 bp, respectively. The aligned reads were sorted by chromosomal coordinates using Samtools [13] and then scored with HTseq-count [14] using the union mode for overlapping reads and the UCSC hg19 gene annotation file.

### Differential expression analysis

All statistics and images were performed in R, version 3.0.1. [15]. A count matrix of all read counts was created from all sequenced samples. Differential expression and multivariate analysis was performed using DESeq2 [16]. This software was also used to create a matrix of normalized expression for each gene by calculating fragments per million mapped fragments (FPM). Utilizing the RNASeqPower package [17] in R, we estimated that for alpha of 0.05 and power (1-beta) of 0.8, with an estimated biological variation coefficient of 0.5 (humans), and around 30 cases of poAF, we could identify an expression difference of more than 50% for genes with more than 25 covering reads per gene, and an expression difference of more than 100% for genes with more than 10 covering reads per gene. Adjustment for multiple testing was done by calculating False Discovery Rate (FDR) using the method of Benjamini-Hochberg [18]. Genes with FDR-adjusted *p*-value less than 0.1 and absolute log2 ratio fold change more than 0.5 were considered significant.

### Functional network analysis

A left atrium co-expression network was generated by calculating the Pearson correlation coefficient between the normalized gene expression values for each gene in each patient. Genes with absolute correlation coefficient of more than 0.8 were considered to be co-expressed. The GeneMANIA server (www.genemania.org) was used to identify similarity between differentially expressed genes, predict their function, and list genes with similar functions [19]. The GeneMANIA algorithm was set to use the left atrium co-expression network in addition to its default co-localization, genetic interaction, pathway, physical interactions, shared protein domains, and predicted gene interaction networks under the automatic weighting method.

### eQTL analysis

For data visualization and expression of quantitative trait loci (eQTL) analysis the normalized gene expression matrix was used. The eQTL analysis was done using the Matrix eQTL package in R [20]. For each SNP, this tests the association between the additive counts of the minor allele and the normalized expression of each gene. Results were focused on genes and SNPs in *cis* eQTL relationship, where the distance between the center of the gene and the SNP is less than 1 megabase. We considered eQTL associations with FDR adjusted *p*-value of less than 0.05 to be statistically significant.

The association between the eight SNPs chosen for genotyping in the cohort of 1,805 patients and poAF was analyzed using PLINK, v1.07 [21]. This was done with an additive genetic model after adjustment for age, gender, bypass time, and if aortic valve replacement was done. A *p*-value of less than 0.05 was considered statistically significant in this analysis.

## Results

### Patient demographics

In our study, 21 out of 62 (34%) patients experienced post-op atrial fibrillation after mitral valve surgery. These patients were older and had a higher frequency of hypertension and statin usage (Table 1) compared to patients who did not experience poAF. Hypertension and statin usage were not significantly associated with poAF after correcting for age (data not shown). Although LA was substantially enlarged in both groups (maximum LA volume of 71 mL/m^2^, compared to the normal range of 15-41 mL/m^2^), [22] there was no difference between patients with and without poAF. Similarly, left ventricular ejection fraction was similar between the two groups (Table 1).

**Table 1:** Patient demographics

	All (*n* = 62)	No poAF (*n* = 41)	poAF (*n* = 21)	*p-*value
Female gender	27 (44%)	17 (41%)	10 (47%)	0.85
Age (mean)	58.7	56.0	64.8	0.006
Past Medical History				
Smoking (Any)	21 (32%)	12 (29%)	8 (38%)	0.68
Myocardial Infarction	2 (3%)	1 (2%)	1 (5%)	1.00
Hypertension	34 (55%)	18 (44%)	17 (81%)	0.01
Diabetes	7 (11%)	4 (11%)	3 (15%)	0.94
Atrial fibrillation	12 (19%)	7 (17%)	5 (24%)	0.76
Mitral Stenosis (severe)	5 (8%)	3 (8%)	2 (11%)	0.65
Mitral Insufficiency				
None	1 (2%)	0 (0%)	1 (5%)	
Mild	1 (2%)	1 (2%)	0 (0%)	
Moderate	5 (8%)	4 (10%)	1 (5%)	
Severe	55 (89%)	36 (88%)	19 (90%)	0.48
Preoperative medications				
ACE inhibitor /ARB	27 (44%)	16 (39%)	12 (57%)	0.28
β-blockers	29 (47%)	18 (44%)	10 (48%)	0.99
Calcium channel inhibitors	8 (13%)	7 (17%)	1 (5%)	0.38
HMG-CoA reductase inhibitor	19 (31%)	9 (21%)	10 (50%)	0.05
Perioperative information				
Left ventricular ejection fraction	0.60	0.61	0.59	0.39
Left atrium volume (ml/m^2^)	71	70	73	0.75
Cross-clamp time (min)	106	108	101	0.58
Cardiopulmonary bypass time (min)	144	146	143	0.85

Patient characteristic of individuals included in the study. poAF – post-operative atrial fibrillation. ACE – angiotension-converting enzyme ARB – aldosterone II receptor blocker HGM-CoA -hydroxy-3-methylglutaryl-coenzyme

### Expression Profile of the LA

On average, RNA sequencing yielded 49.4 million (range 36.5-61.6 million) paired-end reads per sample and 96.1% (range 93.9-96.9%) of reads were aligned to the human genome by TopHat2. Of those 62.2% (range 45.1-76.9%) of the aligned reads were uniquely assigned to a single location. 21,878 genes had at least one read in at least one patient, with a large range of expression between genes. The mean normalized expression was 45.7 FPM (range 0-15,880; interquartile range 0.3-35.8 FPM). As expected, the most highly expressed genes were associated with core cardiac structural proteins, including cardiac actin-myosin chain (*MYH6, ACTC1, MYL7*) and other cardiac-specific genes including *NPPA*, *MB*, *TTNT2*, *TTN* and *ANKRD1* (data not shown).

### Differential gene expression in patients with poAF

**Fig. 1:**
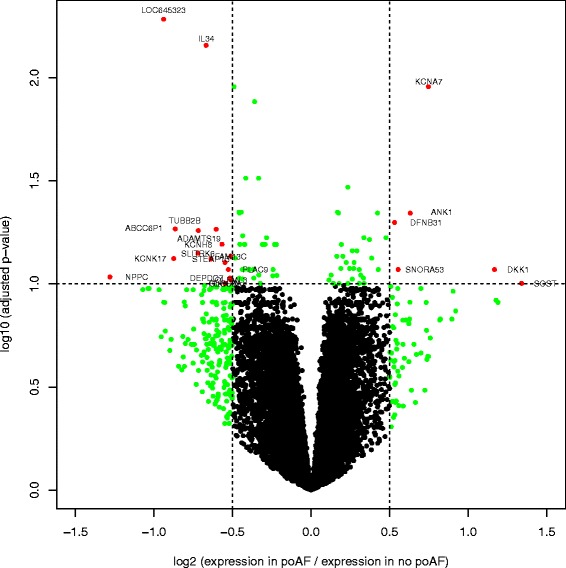
Differentially expressed genes in patients with post-operative atrial fibrillation (poAF). A volcano plot comparing the expression of all genes in the human left atria between patients who had post-operative atrial fibrillation compared to those who did not. The x-axis shows the log2 fold change and the y-axis shows the –log10 of the *p*-value adjusted for multiple testing. Dotted lines mark predetermined levels of significance; absolute log2 ratio >0.5 (x-axis) or *p*-value adjusted for multiple testing <0.1 (y-axis). Green dots indicate genes that fulfill one significance criteria, red dost indicate genes that fulfill both

**Fig. 2:**
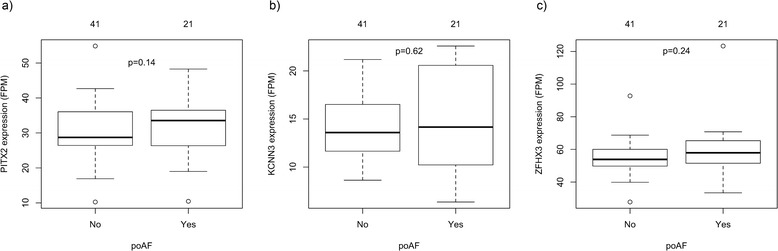
Expression of *PITX2*, *KCNN3,* and *ZFHX3* in the LA of patients with and without post-operative atrial fibrillation (poAF). Boxplots of the normalized expression of **a** *PITX2*, **b** *KCNN3* and **c** *ZFHX3* between patients with and without poAF. The number above each box shows the number of patients in each group. FPM – Fraction per million mapped fragments

We compared the gene expression in the LA between patients who did and did not develop poAF during their hospital stay (Fig. 1). After correcting for known and predetermined predictors of poAF (age, gender, cardiopulmonary bypass time, and LA volume) and accounting for multiple gene testing, we identified 23 differentially expressed genes (Table 2). Of note, we did not see expression changes in genes previously associated with AF by genome-wide association analysis, including *PITX2* [1, 7], *KCNN3* [2, 8], and *ZFHX3* [3] (Fig. 2).

**Table 2:** Genes with differential expression in the left atrium between patients with and without post-operative atrial fibrillation (poAF)

Gene name	Fold change	*p*-value	FDR adj *p*-value
*LOC645323*	-0.9	3 × 10^-7^	0.005
*IL34*	-0.7	8 × 10^-7^	0.007
*KCNA7*	0.7	3 × 10^-6^	0.011
*ANK1*	0.6	4 × 10^-5^	0.045
*DFNB31*	0.5	5 × 10^-5^	0.050
*ABCC6P1*	-0.9	6 × 10^-5^	0.054
*TUBB2B*	-0.6	6 × 10^-5^	0.054
*ADAMTS19*	-0.7	6 × 10^-5^	0.055
*KCNH8*	-0.6	1 × 10^-4^	0.064
*SLITRK6*	-0.7	2 × 10^-4^	0.071
*FAM13C*	-0.5	2 × 10^-4^	0.073
*KCNK17*	-0.9	2 × 10^-4^	0.075
*STEAP1B*	-0.6	2 × 10^-4^	0.076
*RASGEF1C*	-0.5	2 × 10^-4^	0.079
*DKK1*	1.2	3 × 10^-4^	0.085
*PLAC9*	-0.5	3 × 10^-4^	0.085
*SNORA53*	0.6	3 × 10^-4^	0.085
*NPPC*	-1.3	4 × 10^-4^	0.092
*DEPDC7*	-0.5	4 × 10^-4^	0.094
*OLFML3*	-0.5	4 × 10^-4^	0.096
*CLEC9A*	-0.5	5 × 10^-4^	0.099
*PDGFRL*	-0.5	5 × 10^-4^	0.099
*SOST*	1.3	5 × 10^-4^	0.099

Genes were required to fulfill two criteria to be considered differentially expressed, the absolute of the log2 of the ratio of expression in patients with poAF and patients without poAF (fold change) had to be more than 0.5, and the *p*-value for the expression difference adjusted to account for false discovery rate (FDR) was required to be less than 0.1

### Functional analysis of differentially expressed genes in poAF

**Fig. 3:**
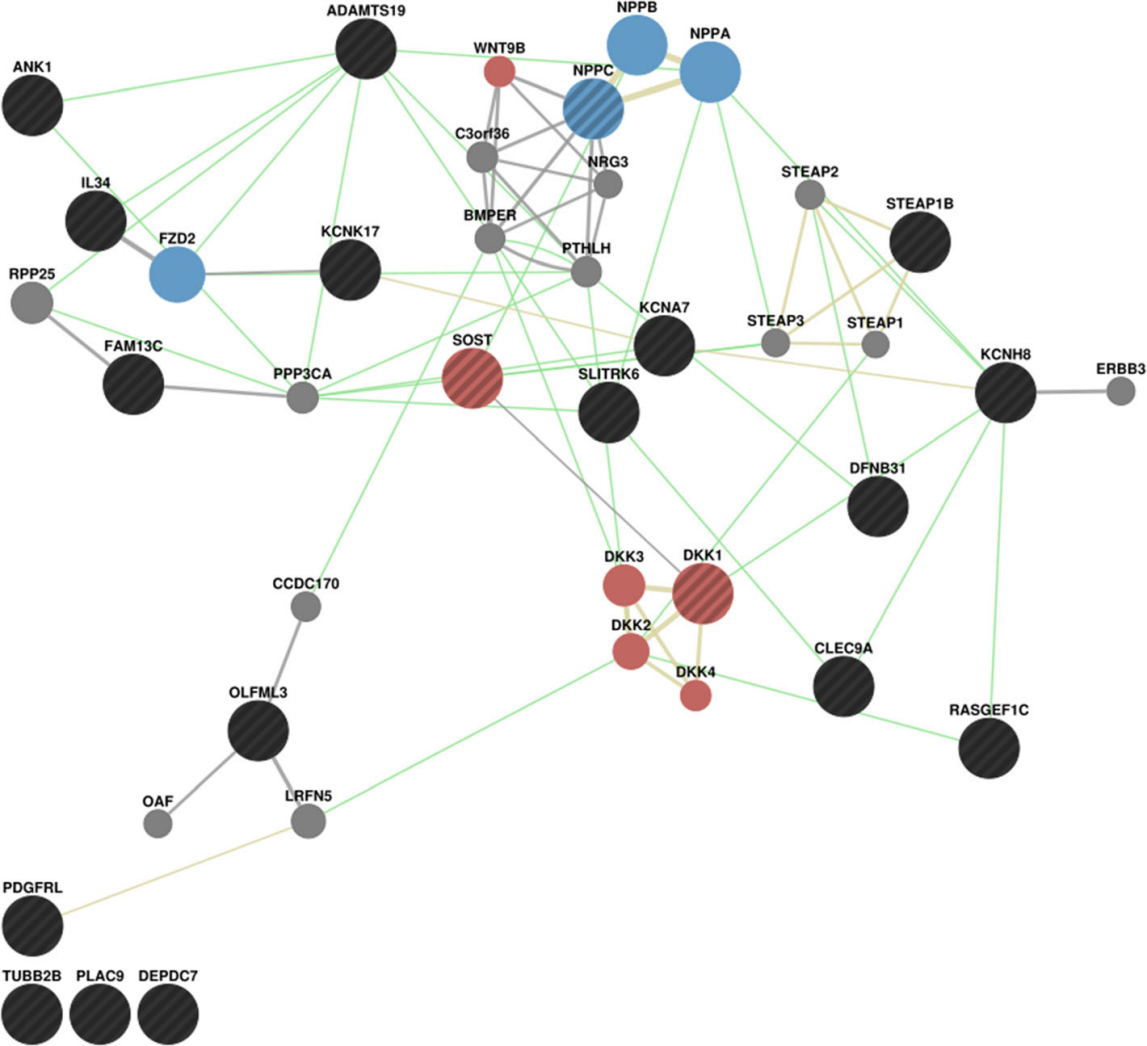
Gene Network Analysis. Result of the functional network analysis of the list of genes with differential expression in poAF by the GeneMANIA algorithm [19]. Connections were assessed using the list of differentially expressed genes (black, black stripes) in the context of a left atrial co-expression network (*gray connections*) and the default GeneMANIA networks on genetic interactions (*green*) or shared protein domains (*yellow*). Two gene function categories were overrepresented in the output, the Wnt Signaling pathway (*red*) and the cGMP metabolic process (*blue*)

The list of differentially expressed genes in was submitted to the GeneMANIA algorithm [19] to identify functional connections. Overrepresentation analysis uncovered enrichment in two distinct functional classes (Fig. 3); the wingless integrated (wnt) signaling pathway (FDR adjusted *p*-value = 0.002) and the cyclic guanosine monophosphate metabolism pathway (FDR adjusted *p*-value = 0.002).

### Expression quantitative trait loci (eQTL) analysis of the left atrium

In 62 patients, mean overall SNP genotyping rate for the ~2.5 million SNPs was 99.5%. For analysis, we used the genotypes of all SNPs with at least 98% genotyping rate, a Hardy-Weinberg equilibrium *p-*value of >10^-5^ and a minor allele frequency of >10%, yielding 1,028,887 SNPs. A genome-wide *cis* eQTL analysis of the human LA was performed comparing the genotypes of all SNPs with the expression of all genes within 1 megabase.

**Fig. 4:**
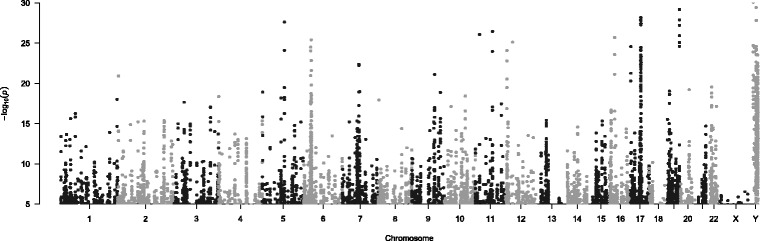
Genomic location of *cis*-eQTL relationships in the human LA. A Manhattan plot of the genomic location of all significant *cis*-eQTL (at false discovery adjusted *p* < 0.05) relationships in the human left atrium

At an FDR adjusted *p*-value < 0.05, a total of 16,139 *cis* eQTL relationships between 13,927 different SNPs and 1,880 different genes were found (Fig. 4, Additional file 1). After exclusion of poorly expressed gene-SNP pairs (<0.1 FPM) and eQTL relationships including the Y chromosome, the strongest *cis* eQTL association was between rs78042921 and the expression of the X-Ray Radiation Resistance Associated 1 (*XRRA1*) gene on chromosome 11, along with other SNPs in high linkage disequilibrium with rs78042921. The highest number of eQTL relationships was found between SNPs and the ring finger protein 5, E3 ubiquitin protein ligase pseudogene 1 (*RNF5P1*) on chromosome 8 (*n* = 748). A cluster of genes on chromosome 6 encoding members of the major histocompatibility complex (MHC) class I including *HLA-DRB5*, *ZFP57*, *MICA*, *MICB*, *ZNRD1-*AS1, *CDSN, HLA-DRB6*, *PSORS1C2* and *HLA-H* were also among the genes with the highest number of *cis* eQTLs.

### eQTL analysis of variants associated with AF by genome-wide association studies

**Fig. 5:**
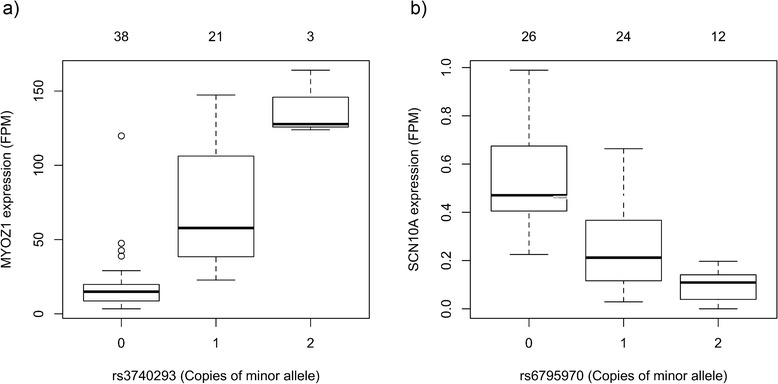
*cis* eQTL relationships of SNPs and genes associated with AF. Boxplots of the normalized expression of each gene (y-axis) and the genotype (allele counts of minor allele) for **a** *MYOZ1* and rs3740293 and **b** *SCN10A* gene and rs6795970. The number above each box shows the number of patients with each genotype. FPM – Fraction fragments per million mapped fragments

The LA eQTL results were analyzed to identify significant *cis* eQTL relationships that included regions within 500 kb of the location of SNPs previously shown to be associated with AF in ambulatory and surgical cohorts [23]. This uncovered 9 such relationships (Table 3), including a strong eQTL association between rs3740293 and *MYOZ1* expression (Fig. 5a, Additional file 1). The genotype of rs3740293 has previously been associated with the expression of the *MYOZ1* gene in left and right atrial tissue [24], and this SNP is in high linkage disequilibrium (LD) with rs10824026, which has also been associated with AF [24, 25].

**Table 3:** cis eQTL relationships involving variants close to regions associated with AF via genome-wide association studies

SNP	Gene	Chromosome	SNP Location	*p*-value	FDR adjusted *p*-value
rs60632610	*MYOZ1*	10	75,415,677	1.7 × 10^-14^	3.5 × 10^-10^
rs10894154	*CNTN5*	11	99,781,954	2.8 × 10^-6^	0.007
rs4437927	*FMO6P*	1	170,196,342	6.2 × 10^-6^	0.01
rs9409797	*MIR27B*	9	97,276,713	6.5 × 10^-6^	0.01
rs6590399	*CNTN5*	11	99,759,525	7.4 × 10^-6^	0.01
rs8847	*RIT1*	1	155,259,323	1.0 × 10^-5^	0.02
rs305043	*PAQR5*	15	70,088,292	1.8 × 10^-5^	0.03
rs10908463	*RIT1*	1	155,286,258	2.2 × 10^-5^	0.03
rs11264341	*GBAP1*	1	155,151,493	3.1 × 10^-5^	0.04

Shown are all significant *cis* eQTL relationships (at FDR-adjusted *p*-value < 0.05) between SNPs previously characterized as being associated with AF and genes within 500 kb [23]. For each gene, the most significant eQTL relationship is shown. Variant location is in coordinates from the UCSC hg19 genome annotation

### eQTL analysis of variants associated with AF by candidate-gene analysis

The LA eQTL results were analyzed to identify significant *cis* eQTL relationships involving genes previously shown to be associated with AF via candidate gene analysis [4, 6]. This analysis identified ten eQTL relationships involving the *SCN10A* gene and adjacent SNPs in high LD (Additional file 1). These included rs6800541, previously associated with AF, and rs6795970, a missense variant in the *SNC10A* gene (Fig. 5b).

### eQTL analysis of variants associated genes with differential expression in the LA of patients with poAF

The LA eQTL results were analyzed for significant cis eQTLs including those genes that were differentially expressed between those patients that did and did not develop poAF in the LA (Table 2). This analysis identified *cis* eQTL relationships involving four out of the 23 differentially expressed genes (Table 4). These SNPs could potentially be novel candidate markers involved in AF or poAF.

**Table 4:** *cis* eQTL relationships of genes differentially expressed in poAF

SNP	Gene	Chromosome	Location	*p*-value	FDR adjusted *p*-value
rs10759721	*DFNB31*	9	117,326,038	3.7 × 10^-7^	0.001
rs4802546	*KCNA7*	19	49,570,686	9.4 × 10^-6^	0.02
rs4911071	*SLITRK6*	13	85,911,610	1.6 × 10^-5^	0.03
rs2393581	*FAM13C*	10	61,801,282	3.3 × 10^-5^	0.04

Significant *cis* eQTL relationships (at FDR-adjusted *p*-value <0.05) between genomic variants and genes with differential expression in our poAF study (Table 2). For each gene, the most significant eQTL relationship is shown. Variant location is in hg19 coordinates

### Association of novel SNPs with poAF in a larger cohort

We tested whether variants with eQTL relationships with differentially expressed genes in the LA of our surgical patients were also associated with poAF in a larger cohort of patients undergoing cardiac surgery (Table 5). This was accomplished by genotyping four SNPs with such associations in an additional cohort of 1,805 Caucasian individuals undergoing heart surgery (Table 5). One out of four SNPs, namely rs10759721, was found to be associated with poAF in this larger cohort. This SNP has an eQTL relationship with the expression of the *DFNB31* gene. The *DFNB31* gene was more highly expressed in the LA of patients with poAF, and the eQTL analysis identified a negative correlation between the expression of the *DFNB31* gene and the number of minor alleles (T) of rs10759721. Therefore the minor allele may be associated with protection against poAF. This relationship matches the results from the larger cohort, where individuals with the minor allele had reduced odds of poAF (OR 0.81 per minor allele (T), 95% CI 0.69-0.95, *p* = 0.01).

**Table 5:** Association of candidate SNPs with poAF in a large independent cohort of surgical patients

SNP	Associated Gene	OR	95% CI	*p*-value
rs10759721	*DFNB31*	0.81	0.69-0.95	0.0100
rs10817638	*DFNB31*	0.84	0.71-0.99	0.0415
rs60632610	*MYOZ1*	1.12	0.90-1.39	0.31
rs4802546	*KCNA7*	1.00	0.78-1.29	1.00

4 SNPs identified as having an association with poAF in the eQTL study were validated in an independent cohort of 1805 surgical patients. Shown is the odds ratio, 95% CI, and unadjusted p-value for the association, after adjustment for age, gender, bypass time and the performance of valve surgery

## Discussion

This study analyzed human LA gene expression differences and eQTL relationships in the human LA, emphasizing expression differences and eQTL relationship affecting atrial fibrillation. Several authors have studied the gene expression profile of the human LA in the context of comparing it to the profile of the right atrium, and also comparing right atrial or right atrial appendage expression profiles of patients with and without poAF [24, 26–28]. Nevertheless, the limitations of these other studies include small patient numbers, the use of the left or right atrial appendage as a surrogate for LA tissue, and the inherent complications of older technological approaches such as microarrays for expression profiling.

The most significant differentially expressed gene identified, *LOC645323*, is a long noncoding RNA known to be highly expressed in neuronal tissue. Transgenic knockout mice of this gene resulted in no identifiable phenotype, and no other studies describing its function in humans have been reported [29]. This gene however resides in a highly conserved region that includes the miRNA miR-92, and myocyte enhancer factor 2c (*MEF2C*) gene, a myocardial transcription factor whose protein levels are associated with arrhythmias in mice [30]. Long noncoding RNA molecules affect both local and distal gene transcription, so it is possible that the expression of *LOC645323* influences the expression of the adjacent the *MEF2C* gene, resulting in the observed poAF phenotype.

From the list of genes with differential expression between patients with and without poAF there were several involving potassium channels (Table 3). In addition to prior association of potassium channel genes (*KCNN3, KCNA5* and *KCNE1-5*) with AF described by others [4], we identified three additional genes in the potassium channel gene family with differential expression in poAF. The *KCNA7* gene encodes a voltage-gated potassium channel [31] the *KCNH8* gene encodes a voltage-dependent potassium channel [32], and the *KCNK17* gene codes for a potassium channel that contributes to the resting membrane potential [33]. None of these genes has thus far not been associated with an arrhythmia. Interestingly, decreased left atrial expression of the *PITX2* gene in mice was found to be associated with lower resting membrane potential via modification of background potassium currents [34]. Furthermore, a lower resting membrane potential was associated with increased sensitivity to sodium channel blockers to prevent AF in the experimental animals. These findings and ours suggest that there might be a subset of patients with AF whose arrhythmia is caused by altered resting membrane potential mediated by potassium currents.

Similar to a recent study [35], we did not observe differences in the *PITX2* expression between subjects with and without poAF. This indicates that the identified association between DNA variants within these genes and AF are not mediated via overall expression changes, although it is possible that this also reflects differences between the study population of patients with mitral valve disease and ambulatory populations with AF in the absence of mitral valve disease.

Network analysis identified that the list of differentially expressed genes was enriched in two functional pathways, namely Wnt signaling and cGMP metabolic process. Wnt proteins are intracellular signaling molecules that trigger distinct canonical and noncanonical pathways. In the canonical pathway, binding of Wnt proteins at the plasma membrane triggers a phosphorylation cascade that stabilizes β-catenin, a nuclear co-activator of several transcription factors [36]. This pathway plays a significant role in early cardiac development, where it regulates the generation of mesoderm formation and cell adhesion in cardiomyocytes [37]. It has been suggested that reactivation of Wnt signaling in the aging heart contributes to cardiac fibrosis, which may contribute to a further risk of AF [38]. More recently, analysis of Pitx2 loss-of function mouse models revealed that complex relationships exist between the expression of the Pitx2 gene and calcium homeostasis via Wnt signaling [39]. The authors believe that this mechanism could be responsible for the association between the *PITX2* gene and AF. The results of our study further support involvement of the Wnt signaling pathway in the pathogenesis of AF in humans, potentially through cardiac fibrosis and its effect on calcium current homeostasis.

The cGMP pathway is involved in several signaling pathways including nitric oxide (NO) mediated cardiomyocyte ion-channel function. Indeed, platelet cGMP levels, used as a surrogate assessment for the activity of the NO pathway, were found to be reduced in patients with AF compared to patients in sinus rhythm [40]. In addition to its role in regulating vascular tone, NO also effects cardiac ionic currents, including potassium currents regulating the resting membrane potential, and thus may play a role in the pathogenesis of AF [41].

Several of the *cis* eQTL relationships identified, involve the major histocompatibility complex (MHC). Furthermore, the *UTY* gene, the strongest eQTL relationship, is also a minor histocompatibility complex gene. These findings are similar to several eQTL studies from other tissues that identify MHC as a major eQTL source [42]. A cautious interpretation of eQTLs including the MHC regions has been encouraged, as the extreme variability of this region can bias expression quantification of microarray and sequencing methods towards detecting the reference genome sequence. This can render individuals with many minor alleles of MHC genes less likely to have detectable expression of the MHC genes, creating spurious eQTL relationships [42].

Among the strongest *cis* eQTL relationships involving regions associated with ambulatory AF were relationships between variants on 10q22 including rs3740293 and the *MYOZ1* gene (Table 3, Fig. 5). Lin et al. also identified a *cis* eQTL relationship between rs3740293 and the expression of *MYOZ1* with microarray and quantitative real-time polymerase chain reaction [24]. Previously, the SNP rs10824026 in this locus has been strongly associated with AF [25]. This SNP is in high LD with many of the SNPs we identified to be associated with *MYOZ1* expression, including rs3740293. The *MYOZ1* gene is expressed in heart and skeletal muscle and is involved in calcineurin signaling which establishes a stable sarcomere. Based on our eQTL findings, it is possible that the identified relationship between rs3740293 and ambulatory AF, stem from altered expression of this gene in minor allele carriers, resulting in altered sarcomere mechanics and calcium homeostasis contributing to poAF [43].

Several *cis* eQTL relationships were found involving the expression of the *SCN10A* gene. The *SCN10A* gene encodes a voltage-gated sodium channel Na_v_1.8 [44], and variants in this gene have been associated both with variations in the PR interval and with AF [45]. The rs6795970 SNP leads to a missense mutation in *SCN10A* and is in a high LD with rs6800541, which resides in one of its introns. Recently, rs6795970 was found to be protective against AF in a lone AF cohort [6]. Functional studies with the rs6795970 variant in cell culture have revealed increased peak and sustained currents, and slowing of fast inactivation of the cell membrane [6]. Our results indicate that the membrane electrophysiological properties associated with the SNP may be mediated by decreased expression of the *SCN10A* gene or the expression of the *SCN10A* gene is decreased as a response to this gain-of function mutation.

Several limitations of the study should be mentioned. Any duration of arrhythmia was included in in our definition of poAF. This was done under the understanding that although poAF of short duration might not have a clinical relevance it likely indicates a genetic predisposition towards the arrhythmia. We sampled tissue from the LA free wall, which is structurally and functionally more similar to the tissue involved in the pathophysiology of AF. This however limited the number of patients included, and the amount of available tissue. The small amount of tissue available precluded us from performing independent biological replicates to minimize variability in RNA expression estimates. Furthermore, the study cohort of patients presenting for mitral valve surgery limits the generalizability of the results to other populations. In particular, the larger cohort used to test the association of SNPs in eQTL relationship with differentially expressed genes stems from a surgical cohort undergoing aortic valve and coronary bypass surgery. Finally, the expression changes observed in poAF might not translate completely to ambulatory AF, as they represent two different processes. We do however think that some of the genetic predisposition towards poAF is shared with ambulatory AF. This is supported by the ability of our group to correlate genetic variants associated with ambulatory AF to poAF after cardiac surgery using the same definition of poAF of any duration [7].

## Conclusion

In summary, we describe the expression landscape of the human LA with a focus on those genes involved in poAF. It is likely that either poAF or ambulatory AF represent a final common phenotype of alteration in multiple different electrophysiological pathways. We found association between poAF and both the resting membrane potential as well as pathways associated with calcium homeostasis and atrial fibrosis. The results of the eQTL analysis further the understanding of how variants associated with AF and poAF mediate their effects, in particular how variants adjacent to the *MYOZ1* and *SCN10A* genes might modulate their effects on AF risk. The genetic signatures of poAF and ambulatory AF have thus far proven to be highly similar despite different environmental insults associated with the onset of the phenotype. Therefore, it is our hope that our results can further both the understanding of AF pathophysiology in both post-surgical and ambulatory populations.

## Additional file

Additional file 1: All *cis* eQTL relationships in LA at false discovery rate (FDR)-adjusted *p*-value <0.05. Location is in hg19 coordinates. Mean gene expression is in normalized FPM (fragments per million mapped fragments). (TXT 2516 kb)

## Abbreviations

AF: Atrial fibrillation
EQTL: Expression quantitative trait loci
FDR: False discovery rate
LA: Left atrium
PoAF: Post-operative atrial fibrillation
SNP: Single nucleotide polymorphism
